# Soy isoflavones ameliorate the cognitive dysfunction of Goto-Kakizaki rats by activating the Nrf2-HO-1 signalling pathway

**DOI:** 10.18632/aging.103877

**Published:** 2020-11-07

**Authors:** Boxi Ke, Tianmeng Zhang, Tianyang An, Rong Lu

**Affiliations:** 1 Department of Neurology, Taizhou Central Hospital (Taizhou University Hospital), Taizhou, Zhejiang Province, PR China; 2 Anesthesiology Department, Taizhou Central Hospital (Taizhou University Hospital), Taizhou, Zhejiang Province, PR China; 3 Jitang College of North China University of Science and Technology, Tangshan, Hebei Province, PR China

**Keywords:** cognitive decline, soy isoflavones, ROS, Nrf2-HO-1 signal pathway

## Abstract

Soy isoflavones (SIF) are soybean phytochemicals that are considered to be biologically active components that protect from neurodegenerative diseases. In this study, the therapeutic effect of SIF was evaluated in a diabetic Goto-Kakizaki (GK) rat model. Twenty male GK rats were randomly divided into diabetes mellitus (DM) model group and SIF+DM group (n=10 in each group). Twenty age-matched male Wistar rats were randomly divided into control group (CON group) and CON+SIF group, with 10 rats in each group. The learning and memory functions of the animals were determined by the Morris water maze (MWM) test. Hematoxylin-eosin staining (HE) was performed to examine pyramidal neuron loss in the CA1 area of the hippocampus. Markers of oxidative stress (OS) were measured to evaluate oxidative stress-mediated injury. RT-PCR and western blotting were used to analyze the expression of nuclear factorerythroid2-related factor 2 (Nrf2), heme oxygenase-1 (HO-1) and NAD(P)H dehydrogenase quinone1 (NQO1). Treatment with SIF for 4 weeks alleviated the cognitive dysfunction of the GK rats as determined by the MWM test. Moreover, SIF treatment also reduced diabetes-related oxidative reactions. In addition, SIF enhanced the expression of Nrf2, HO-1 and NQO1, suggesting a potential antioxidation mechanism for the effect of SIF. These findings suggest that SIF can be considered candidates for inhibiting the progression of diabetes-induced cognitive dysfunction, provide novel insights into the antioxidant effect of SIF and further strengthen the link between oxidative stress and diabetes.

## INTRODUCTION

Diabetes is a kind of endocrine disease that is attracting attention worldwide, and the incidence of diabetes in China is increasing every year [1, 2]. There are many complications that are caused by diabetes and affect multiple organs, including damaging both the peripheral and central nervous systems. There is strong evidence that DM is a risk factor for cognitive dysfunction, including Alzheimer's disease (AD) [3, 4]. However, the exact pathological mechanism of cognitive dysfunction in diabetes patients is not well understood. The hippocampus is an important area of the brain that is related to learning and memory, and recent evidence has indicated that sustained hyperglycemia can damage the nerve cells in the hippocampus [5, 6]. DM is a chronic metabolic disease that is associated with oxidative stress, which is an important risk factor that leads to cognitive dysfunction [7]. Hyperglycemia-mediated oxidative stress plays an important role in the progression of cognitive decline [8, 9]. Nrf2-mediated endogenous antioxidant expression is thought to be an important mechanism of the body's defense against DM-induced oxidative stress (OS)-mediated damage [10]. To a certain extent, excessive ROS may promote the occurrence of DM and its related diseases. In the high-glucose state of DM, intracellular glucose oxidation is increased, and mitochondria produce excessive superoxide, causing OS-mediated damage to cells in tissues. OS can cause IR by activating OS-related pathways. Activation of the Nrf2-ARE pathway can reduce the OS-mediated damage caused by diabetic hyperglycemia [11]. The accumulation of oxidative stress products has serious effects on memory dysfunction and damages hippocampal neurons [12]. Currently, there is no effective drug for the treatment of cognitive dysfunction. Therefore, effective treatment is urgently needed to improve cognitive impairment and prevent AD development.

Soy isoflavones (SIF) are effective ingredients of soy and are generally considered to be beneficial. Soy has been a regular part of the diet in nearly all of the countries in the world for centuries, and the consumption of soy can lower the incidences of cardiovascular disease and the risk of ischemic stroke [13, 14]. Recent evidence has indicated that SIF are considered to be the active ingredients that exert beneficial effects against neurodegenerative diseases [15–18]. Clinical studies have shown that SIF can ameliorate cognitive dysfunction in elderly adults [19].

In the current study, we used the diabetic GK rat model, which is a commonly used model of diabetes [20, 21], to investigate the effects of SIF on the memory decline in diabetic rats. Accordingly, the study aimed to identify possible mechanisms by which SIF may benefit cognitive function.

## RESULTS

### Effects of SIF on weight and blood glucose in diabetic GK rats

**Figure 1:**
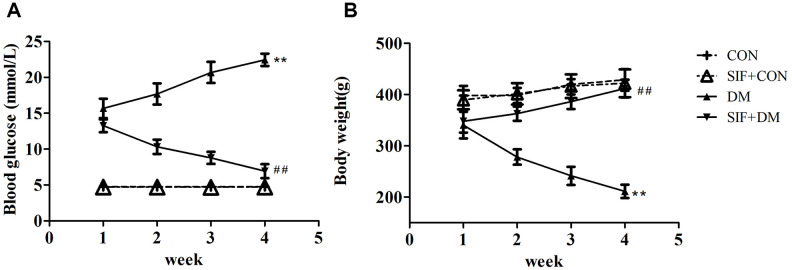
**Effect of SIF on the body weights and blood glucose levels of diabetic rats.** (**A**) Changes in the blood glucose levels in the rats after four weeks of SIF treatment. (**B**) Changes in the body weights of the rats after four weeks of SIF treatment. The results are shown as the mean±standard deviation (n=10 per group). **p < 0.01 vs. the CON group, ^##^p <0.01vs. the DM group.

We measured the blood glucose levels and weights of the rats (Figure 1). The results showed that the SIF reduced the blood glucose levels and increased the weights of the diabetic rats, suggesting that SIF could effectively improve the blood glucose levels and weight loss of diabetic rats.

### Effects of SIF on cognitive deficits in diabetic GK rats

**Figure 2:**
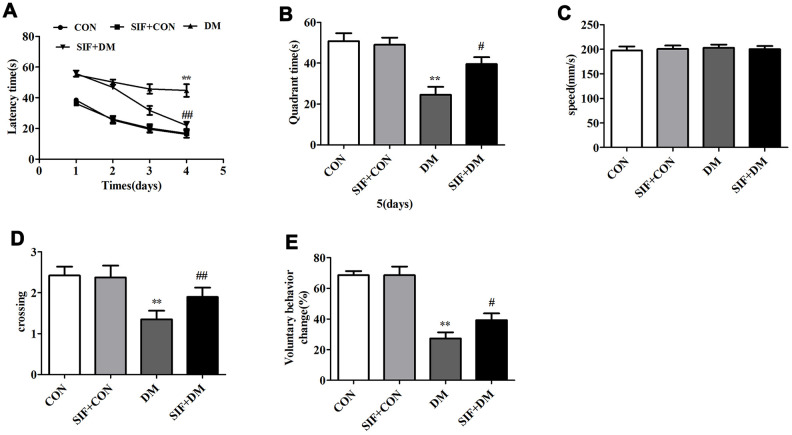
**Effect of SIF on the spatial memory of diabetic rats.** (**A**) Time (seconds) spent finding the platform submerged under the water at days 1-4; (**B**) Exploration time (seconds) spent in the target quadrant that contained the platform at day 5; (**C**)The rats in all three groups displayed no difference in swim speed. (**D**) The number of times the rats crossed the target quadrant. (**E**) The percentage of self-changing behavior in the Y maze of the rats. The results are shown as the mean±standard deviation (n=10 per group). *p < 0.05 or ^##^p <0.01vs. the CON group, ^#^p <0.05 or ^##^p <0.01 vs. the DM group.

The hippocampus-dependent cognitive abilities of the rats were tested by the MWM hidden platform task. Figure 2A shows the effects of SIF administration on the learning and memory abilities of the rats during latency trials. The rats in the DM group spent a longer time searching for the hidden platform during days 1-4 of the latency trials. However, the rats in the SIF+DM group spent obviously less time searching for the platform than the rats in the DM group. On day 5, in the probe test, the platform was removed, and the rats in SIF+DM group spent less time in the target quadrant than the rats in the CON group. The SIF-treated rats spent more time in the target quadrant (Figure 2B). We also measured the number of times the rats crossed the platform, and the crossing times in the DM group were lower than those in the CON group. We also found that the rats in the SIF+DM group crossed the platform more frequently (Figure 2D). Nevertheless, in the whole test, all the rats’ swimming speeds were not significantly different (Figure 2C). As shown in Figure 2E, the voluntary behavior change of the GK rats was significantly lower than that of the CON rats; however, SIF reversed this phenomenon. The behavioral results indicated that the learning and memory abilities of the diabetic rats were significantly impaired, and SIF contributed greatly to the improvement of the learning and memory impairment in the GK rats.

### SIF prevent neuronal cell loss in the CA1 region of the hippocampus

**Figure 3:**
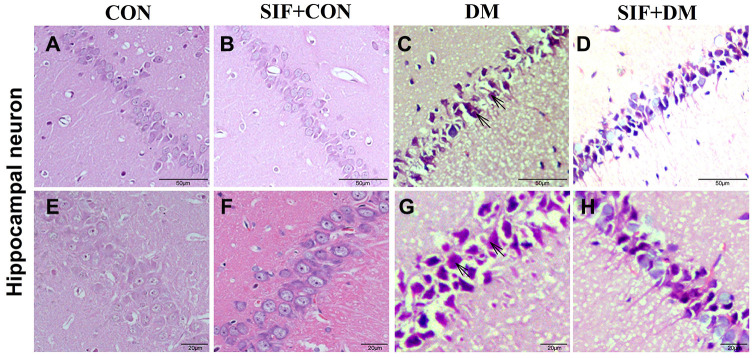
**SIF inhibited neuron loss after 4 weeks of treatment.** HE staining was performed on the sections of the rat hippocampus. (**A–D**) Representative hippocampal photomicrographs of the four groups, 4x, scale bar = 50 μm; (**E–H**) Representative hippocampal photomicrographs of the four groups, 40x, scale bar = 20 μm. The arrows indicate positive signs of hippocampal neurons (shrunken or irregular shape and deeper staining).

We used HE staining to investigate the morphologically intact neurons in the CA1 region of the hippocampus. In the hippocampus, compared to the CON group, the DM group exhibited a greater number of pyramidal cells with shrunken or irregular shapes and deeper staining (Figure 3C, 3G). Treatment with SIF reduced the number of dead cells (shrunken or irregular shape and deeper staining) in the rats with diabetes (Figure 3D, 3H). Our results showed that the morphology of hippocampal neurons in the GK rats was significantly abnormal, and the morphological abnormalities of these hippocampal neurons were improved by SIF administration, suggesting that the improvement of cognitive impairment by SIF was achieved by improving the abnormalities of the hippocampal neurons in GK rats.

### Effects of SIF on oxidative stress markers

**Figure 4:**
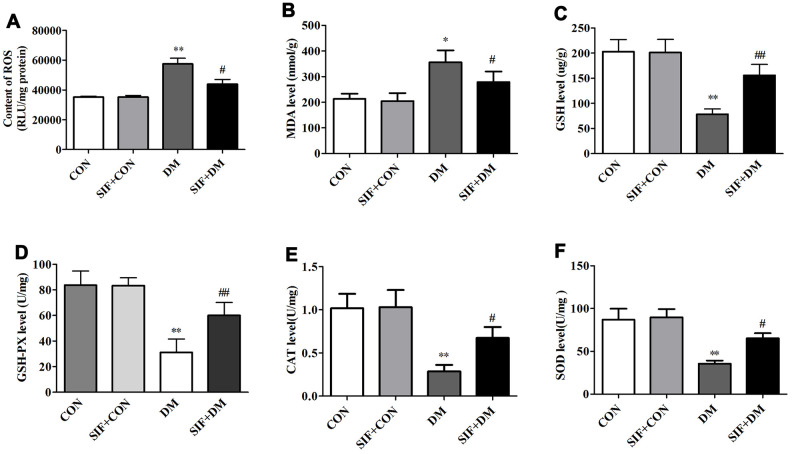
**Effects of SIF on oxidative stress markers.** (**A**) Level of ROS; (**B**) Level of MDA; (**C**) Level of GSH; (**D**) Level of GSH-PX; (**E**) Level of CAT (**F**) Level of SOD. The results are shown as the mean±standard deviation (n=5 per group). *p < 0.05 or ^##^p <0.01vs. the CON group, ^#^p <0.05 or ^##^p <0.01 vs. the DM group.

To explore the mechanism underlying the neuroprotective effect of SIF, we measured the level of ROS and the oxidative stress markers MDA, GSH, GSH-PX, CAT and SOD in the hippocampus of the experimental animals. As shown in Figure 4A, the ROS levels in the rats in the DM group were higher than those in the rats in the CON group. However, the level of ROS significantly decreased after SIF treatment. The level of MDA was significantly increased (Figure 4B) and the levels of GSH, GSH-PX, CAT and SOD was significantly decreased in the hippocampus of the DM rats (Figure 4C–4F). Post treatment with SIF could ameliorate the DM-induced oxidative stress while decreasing the level of MDA (Figure 4B) and increasing the levels of GSH, GSH-PX, CAT and SOD in the hippocampus (Figure 4C–4F). Our results suggest that the cognitive impairment of diabetic rats may be related to oxidative stress, which is consistent with previous studies [24]. After SIF treatment, the indicators of oxidative stress in the diabetic rats showed a positive trend, suggesting that SIF may improve the cognitive dysfunction of diabetic rats by reducing oxidative stress.

### Effects of SIF on the diabetes-induced changes in n-Nrf2, Nrf2, HO-1, and NQO1

**Figure 5:**
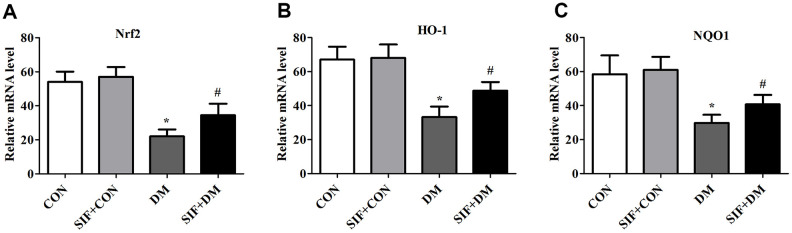
**Effects of SIF on oxidative stress genes.** Relative mRNA expression of Nrf2(**A**), HO-1(**B**) and NQO1(**C**) in the hippocampus. The results are shown as the mean ±standard deviation (n=5 per group). *p < 0.05 or **p < 0.01 vs. the CON group, ^#^p <0.05 or ^##^p <0.01 vs. the DM group.

**Figure 6:**
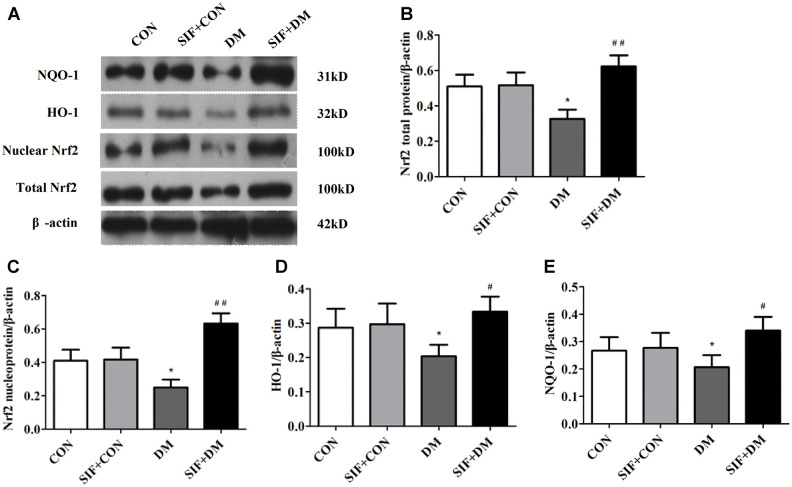
**Effect of SIF on the expression of proteins associated with oxidative stress.** (**A**) Immunoblot bands of Nrf2, HO-1, NQO1 and n-Nrf2; Analysis of each band by optical density. (**B**) Nrf2, (**C**) n-Nrf2, (**D**) HO-1, (**E**) NQO1. The results are shown as the mean ±standard deviation (n=5 per group). *p < 0.05 or **p < 0.01 vs. the CON group, ^#^p <0.05 or ^##^p <0.01 vs. the DM group.

To explore whether Nrf2-ARE signalling was involved in the DM-induced oxidative stress, we measured the mRNA levels of Nrf2, HO-1, and NQO1 in the hippocampus. The Nrf2, HO-1, and NQO1 levels were dramatically increased in the hippocampus of the DM rats. However, this effect was substantially inhibited by SIF treatment (Figure 5). Consistent with the RT-PCR results, the protein expression of n-Nrf2, Nrf2, HO-1, and NQO1 was decreased in the hippocampus in the DM group, and the expression of these proteins was greatly increased by post treatment with SIF (Figure 6). The results suggest that the signalling pathway may be involved in the SF-mediated improvement of the cognitive impairment of diabetic rats.

## DISCUSSION

Diabetes mellitus (DM) is one of the most common metabolic diseases, and its morbidity is increasing. On the other hand, diabetes can cause many complications, and these complications may result in disability and compromise the quality of life. Diabetes is a systemic disease, as it affects various body systems to some extent. For example, diabetes can disrupt the proper function of the cardiovascular, gastrointestinal, immune and nervous systems. There is strong evidence that diabetes is a major risk factor for cognitive impairments [25]. With the progression of the disease, patients may develop dementia. However, our knowledge about the pathogenesis of diabetic cognitive dysfunction is very limited. Therefore, there is an urgent need to determine the pathological mechanism and to develop new drugs to attenuate diabetic cognitive impairment.

Soybean as a legume that is rich in indigenous isoflavones. Consumption of soy-containing foods may reduce the risk of ischemic c stroke, cholesterol levels and cardiovascular disease [26]. A large body of evidence suggests that SIF could be biologically active components that enhance memory in disease models [27].

In this study, we evaluated the neuroprotective effect of SIF on the cognitive dysfunction of diabetic rats using the MWM test and Y-maze test. Additionally, to elucidate the underlying mechanism of the effect of SIF, we examined oxidative stress biomarkers and the expression of OS-related proteins in the hippocampus of rats.

First, we detected the blood glucose levels and body weights of the rats, and we unexpectedly observed that SIF could reduce the blood glucose levels and increase the body weights of the diabetic rats, suggesting that SIF could improve the blood glucose levels and body weights of diabetic rats.

The behavioral results showed that the escape latency period of the diabetic rats was significantly longer than that of the normal rats, and the frequency of platform penetration was significantly lower than that of the normal rats. These results suggest that the diabetic rats developed spatial learning and memory impairment. The MWM task data also showed that the rats treated with SIF exhibited obvious increases in the numbers times they crossed the platform location and decreases in their escape latency compared with the DM rats. In the new object recognition experiment, we obtained the same result as the water maze experiment. Our findings were similar to the previously reported effects of SIF in cognitive improvement in a AD model [28]. Taken together, the behavioral tests showed that SIF could attenuate the cognitive deficits in the diabetic GK rats. In parallel to the results of the behavioral test, SIF significantly reduced the loss of neural cells in the CA1 region of the hippocampus. This observation suggested that SIF played a very important role in neural protection in diabetic rats.

Reactive oxygen species (ROS) was regarded as detrimental molecules that cause a variety of chronic disorders, including diabetes mellitus, and growing evidence indicates that ROS can act as secondary messengers and play roles in intracellular signal transmission. Changes in the ROS and oxidant levels in diabetes are more likely biological features than causative factors [29, 30]. It has been reported that oxidative stress is one of the important factors involved in neurodegenerative diseases [31]. There is mounting evidence that learning and memory abilities are associated with the content of oxidative stress products in the hippocampus [32, 33]. Indeed, we observed a cognitive decline in the diabetic rats, which resulted in remarkable oxidative stress-mediated damage. This result is consistent with previous research results [34]. We observed that the oxidative stress marker levels did appear to be effected by SIF.

Oxygen free radicals can cause cellular lipid peroxidation reactions to further generate MDA, and the MDA level in the cell indirectly reflects the metabolism of oxygen free radicals and the degree of cell damage by free radicals [35]. Under physiological conditions, there are natural free radical-scavenging systems in the body, such as glutathione peroxidase (GSH-Px), SOD, and GSH [36]. Under physiological conditions, Nrf2 and Keap1 in the cell combine to block the activity of Nrf2; under oxidative stress conditions, the Nrf2 and Keap1 complex dissociates, and Nrf2 leaves the cytoplasm to enter the nucleus. In the nucleus, Nrf2 combines with ARE, initiates the expression of phase II detoxification enzyme and antioxidant enzyme genes that are regulated by ARE, and promotes the resistance of cells to oxidative stress and nucleophilic compounds. The antioxidant genes regulated by ARE encode proteins, including HO-1 and GSH [37]. These molecules can protect the body from reactive oxygen species-mediated damage [37]. Nrf2 activation induces the synthesis of a series of antioxidant enzymes (including HO-1) to promote unbalanced redox reactions, to restore balance, and to reduce free radical damage [38–39]. Compared with that of the CON group, the MDA content of the DM group was significantly increased; in addition the GSH level and the SOD, GSH-px and CAT activities were significantly reduced. After SIF treatment, the MDA content was significantly reduced, the GSH level was significantly increased, and the SOD, GSH-px and CAT activities were significantly increased. The RT-PCR and Western blot test results showed that compared with the DM group, the SIF+DM group exhibited significantly up-regulated Nrf2, HO-1, and NQO-1 protein and mRNA levels, indicating that SIF has antioxidant effects. The mechanism of broken damage may be related to the activation of the Nrf2/HO-1 signalling pathway.

In our study, diabetic rats developed oxidative stress, and oxidative stress activated the Nrf2-HO-1 pathway. However, we obtained the opposite results, which may be because the high-glucose environment in rats damages the Nrf2-HO-1 pathway [40]. In future research, we will further study the relationship between blood glucose concentration and ROS and the Nrf2-HO-1 pathway.

### Ethics statement

The experiment was approved and performed in accordance with the Ethics Committee of the Laboratory Animal Center of Taizhou Central Hospital.

## CONCLUSIONS

In conclusion, our present results clearly showed that SIF improved the memory impairments in a diabetic rat model by inhibiting oxidative stress-mediated damage via the modulation of the Nrf2-HO-1signalling pathway.

## MATERIALS AND METHODS

### Experimental animals

Twenty13-week-old male GK rats and 20 age-matched male Wistar rats were provided by Taizhou Central Hospital (Taizhou University Hospital). The rats were housed in individual wire cages in a controlled environment (25 ± 2°C) with a 12 h light/dark cycle. The animals were allowed free access to standard laboratory chow. The experiment was approved by and performed in accordance with the Ethics Committee of the Laboratory Animal Center of Taizhou Central Hospital. All the rats received humane care in compliance with institutional regulations. After one week of adaption, the GK rats were randomly divided into two groups, namely, the DM model group and SIF+DM model group, and the Wistar rats were randomly divided into two groups, namely, the CON group and SIF+CON group (n=10 in each group). The rats in the SIF +DM and SIF+CON groups were treated orally with SIF (20 mg/kg) once daily for 4 consecutive weeks. The SIF were purchased from Xi'an Zebang Biological Technology Co., Ltd. During the 4 weeks of administration, the body weights and blood glucose levels of the rats were measured.

### Morris water maze test

All the animals were subjected to memory assessment by the MWM test [22]. The MWM had a diameter of 100 cm and height of 35 cm, and it included a circular plastic pool filled with squid-ink water that was maintained at 22±1°C. An escape platform (height, 15 cm; diameter, 5 cm) was submerged 1 cm below the surface of the water. During the training trials, the animals were placed in the water and allowed to remain on the platform for 10s. The entire experiment lasted 5 days, of which the navigation test lasted 4 days. Each animal was trained 4 times a day for 60 s per session, with 30 min intervals between each pair of training sessions. We randomly selected one quadrant as the entry point and placed each rat into the pool at the wall. Then, we recorded the time required for the rat to locate the platform under the surface of the water. If a rat did not find the platform within 60 s, it was gently placed on the platform for 10 s, and the escape latency was recorded as 60s. Then, the spatial exploration test was carried out on the final test day. The platform was removed, and each rat was allowed to swim in the maze for 60 s. The time spent in the target quadrant over the 60 s was recorded as an index of spatial memory in the rats. The escape latency, distance around the plate, swimming speed, and swimming pattern of each rat were monitored by a camera above the center of the pool.

### Y-maze test

The Y-maze is composed of three long, black, plastic arms, with an angle of 120° between each arm. Each arm is 30 cm long, 10 cm wide, and 25 cm high. At the beginning of the experiment, the rats were placed in the center of the Y maze and allowed to freely explore for 5 minutes. Any maze video tracking software (Stoelting Corporation, USA) was used to record and analyze the entry sequence, entry number and entry time of each arm. According to the description of TYPLT et al. [23], the exploration of the three arms by the rats was not repeated to promote "autonomous behavior change", and the percentage of the total number of entries of each arm reflected the strength of the working memory of the rats.

### HE staining

After transcardiac perfusion, the brains of all the animals were removed, post-fixed overnight at 4°C in cold 4% paraformaldehyde, and embedded in paraffin. The paraffin-embedded tissues were cut into sections with thicknesses of 5μm. H&E staining was performed and examined using light microscopy (magnification, 4x and40x; Olympus Corporation, Tokyo, Japan) to estimate the extent of the neuronal damage in the hippocampal CA1 region.

### Measurement of oxidative stress

After the WMW test, the rats were anaesthetized with urethane (2.5 g/kg). The GK rats were sacrificed, and the hippocampal tissues were washed and homogenized on ice with normal saline. The homogenates were centrifuged at 3000 g for 10 min at 4°C, and the supernatants (100 mL) were used for analysis. The MDA, GSH CAT, SOD and GSH-PX levels were measured using the MDA Assay Kit, GSH Assay Kit, CAT Assay Kit, SOD Assay Kit and GSH-PX Assay Kit (Nanjing Jiancheng Bioengineering, Nanjing, China). The whole experiment followed the manufacturer’s instructions. The level of ROS was measured using an ROS Assay Kit. The brain tissue was homogenized under ice-water bath conditions and centrifuged at 1000 rpm for 10 minutes. Then, the supernatant was collected for analysis. The fluorescence intensity was measured at a wavelength of 525 nm. The results are expressed as fluorescence intensity/mg protein (RLU/mg protein).

### Real-time PCR analysis

We used RT-PCR to assess the effect of SIF on the Nrf2, HO-1 and NQO1 gene expression in the hippocampal tissues. The total RNA was extracted from the samples using TRIzol reagent (Invitrogen, USA). RT-PCR was conducted according to the instruction book provided by the manufacturer of the Bio-Rad thermocycler and SYBR green kit (Invitrogen). The relative gene expression was normalized to that of GAPDH, which was used as the internal standard. The sequences of the gene-specific primers used were as follows:Nrf2 5’- GCCCTAAAGAACAGCCAACCAAT-3’(forward) and 5’-TCTCCCAGGACTTGT-CCGC-3’ (reverse); HO-1 5’-GTTCCCAGGACTTGCCCGA-3’ (forward) and 5’-A-CTGCCTTCTGCTTGTTTCGCT-3’ (reverse); NQO1 5’-GGGCACATCAACGTC-ACCCTCT-3’ (forward) and 5’-GATGGCGACTTCTCCCAGATAG-3’ (reverse); and GAPDH 5’-CTAACGGCAAGCTAACTG-3’ (forward) and 5’-AGTGGTGACTCC-GTCCAGAACA-3’ (reverse).

### Western blot analysis

The rats were sacrificed under deep anaesthesia (2.5 g/kg urethane). The hippocampal tissues were isolated. After extracting the total protein, the concentration was determined using the Bradford method. The proteins were subjected to SDS-PAGE with 12% precast gels and then transferred to PVDF membranes, which were blocked for 1 h in 5% non fat dry milk in TBS-T. The following primary antibodies were used at the indicated dilutions: Nrf2 (1:500; Abcam; ab31163; USA), HO-1 (1:200; Abcam; ab13243; USA), and NQO1 (1:200; Abcam; ab28947; USA). The membranes were then probed with HRP-conjugated secondary antibodies for 1 h. The signals were detected using an ECL kit (Abcam; USA) according to the manufacturer’s instructions. The changes in the relative protein expression are presented as the ratio of the integrated optical density of the target protein bands to that of the β-actin protein band.

### Data analysis

The statistical analysis was performed in SPSS 17.0. All the data are presented as the mean and standard error of the mean (SEM). Differences among three or more groups were compared by one-way analysis of variance (ANOVA), followed by post hoc testing for multiple comparisons. *P* values of 0.05 and 0.01 or less were regarded as significant.
